# Prognostic and predictive role of a metabolic rate‐limiting enzyme signature in hepatocellular carcinoma

**DOI:** 10.1111/cpr.13117

**Published:** 2021-08-23

**Authors:** Zhangding Wang, Yao Fu, Anliang Xia, Chen Chen, Jiamu Qu, Guifang Xu, Xiaoping Zou, Qiang Wang, Shouyu Wang

**Affiliations:** ^1^ Department of Gastroenterology The Affiliated Drum Tower Hospital of Nanjing University Medical School Nanjing China; ^2^ Department of Pathology The Affiliated Drum Tower Hospital of Nanjing University Medical School Nanjing China; ^3^ Department of Hepatobiliary Surgery The Affiliated Drum Tower Hospital of Nanjing University Medical School Nanjing China; ^4^ Jiangsu Key Laboratory of Molecular Medicine Medical School of Nanjing University Nanjing China; ^5^ Center for Public Health Research Medical School of Nanjing University Nanjing China

**Keywords:** hepatocellular carcinoma, metabolism, prognosis, rate‐limiting enzymes, signature

## Abstract

**Objectives:**

Abnormal expression of metabolic rate‐limiting enzymes drives the occurrence and progression of hepatocellular carcinoma (HCC). This study aimed to elucidate the comprehensive model of metabolic rate‐limiting enzymes associated with the prognosis of HCC.

**Materials and Methods:**

HCC animal model and TCGA project were used to screen out differentially expressed metabolic rate‐limiting enzyme. Cox regression, least absolute shrinkage and selection operation (LASSO) and experimentally verification were performed to identify metabolic rate‐limiting enzyme signature. The area under the receiver operating characteristic curve (AUC) and prognostic nomogram were used to assess the efficacy of the signature in the three HCC cohorts (TCGA training cohort, internal cohort and an independent validation cohort).

**Results:**

A classifier based on three rate‐limiting enzymes (RRM1, UCK2 and G6PD) was conducted and serves as independent prognostic factor. This effect was further confirmed in an independent cohort, which indicated that the AUC at year 5 was 0.715 (95% CI: 0.653‐0.777) for clinical risk score, whereas it was significantly increased to 0.852 (95% CI: 0.798‐0.906) when combination of the clinical with signature risk score. Moreover, a comprehensive nomogram including the signature and clinicopathological aspects resulted in significantly predict the individual outcomes.

**Conclusions:**

Our results highlighted the prognostic value of rate‐limiting enzymes in HCC, which may be useful for accurate risk assessment in guiding clinical management and treatment decisions.

## Graphical abstract

**Step1**: Gene Ontology (GO) and Kyoto Encyclopedia of Genes and Genomes (KEGG) pathway analyses of differentially expressed genes from HCC animals RNA‐seq data clustered the majority genes in metabolic pathways. **Step2**: By conjointly analyzing the differentially expressed metabolic rate‐limiting enzymes from RNA‐seq data of HCC animal model and TCGA data, 12 transcripts were overlapped (RRM1, SQLE, PCK1, PYGB, G6PD, PLAT, ACSL1, RRM2, UCK2, ASS1, FBP1 and IMPDH1). **Step3**: Univariate and LASSO Cox regression were performed to screen out 3 rate‐limiting enzymes with a significant overall prognosis(i), three HCC cohorts (TCGA training cohort, internal cohort and an independent validation cohort) were used to validate the metabolic rate‐limiting enzyme signature by Uni/Multivariate Cox regression, KM survival, ROC analysis and Nomogram model.

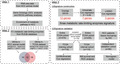

**Abbreviations**
ACSL1: Acyl‐CoA Synthetase Long Chain Family Member 1
ASS1: Argininosuccinate Synthase 1
AUC: area under the ROC curve
CE: cholesterol esters
CMP: cytidine monophosphate
DEN: Diethylnitrosamine
ER: endoplasmic reticulum
FAO: fatty acid oxidation
FBP1: fructose‐1,6‐bisphosphatase 1
G6PD: Glucose‐6‐Phosphate Dehydrogenase
GO: Gene Ontology
HCC: hepatocellular carcinoma
HR: hazard ratio
IHC: Immunohistochemistry
IMPDH1: Inosine Monophosphate Dehydrogenase 1
KEGG: Kyoto Encyclopedia of Genes and Genomes
LASSO: least absolute shrinkage and selection operator
LDs: lipid droplets
NADPH: nicotinamide adenine dinucleotide phosphate
NMIBC: non‐muscle‐invasive bladder cancer.
NSCLC: non‐small cell lung cancer
OS: overall survival
PCK1: Phosphoenol pyruvate Carboxykinase 1
PFKP: phosphofructokinaseplatelet
PLAT: Plasminogen Activator, Tissue Type
PLK3: Polo‐like kinase 3
PPP: pentose phosphate pathway
PPP: pentose phosphate pathway
PYGB: Glycogen Phosphorylase B
qRT‐PCR: Quantitative real‐time RT‐PCR
ROC: receiver operating characteristic
RRM1: Ribonucleotide Reductase Catalytic Subunit M1
RRM2: Ribonucleotide Reductase Catalytic Subunit M2
SOAT1: Sterol O‐acyltransferase 1
SQLE: Squalene Epoxidase
SREBP‐1: Sterol Regulatory Element Binding Transcription Factor 1
TCA: tricarboxylic acid
TCGA: The Cancer Genome Atlas
TMA: tissue microarray
UCK2: Uridine‐Cytidine Kinase 2
UGDH: UDP‐glucose 6‐dehydrogenase
UMP: uridine monophosphate

## 1 INTRODUCTION

Hepatocellular carcinoma (HCC) is a highly aggressive solid malignancy accounting for 90% primary liver cancer and is the fourth leading cause of cancer death worldwide.^1^ Unfortunately, HCC is always diagnosed at advanced stage with extreme hepatic dysfunction, resulting in poor prognosis of HCC patients.^2^ Moreover, current clinical tools to predict prognosis are limited to a set of clinical and pathologic variables, such as the TNM stage, which mainly relies on anatomical information without biological characteristics. Therefore, there is an urgent need to further elucidate the molecular mechanisms of HCC and identify specific prognostic and predictive biomarkers, which would be of great importance to improve the prognosis of HCC patients.

Metabolic reprogramming has been reported to be involved in tumorigenesis and development.^3^, ^4^ Metabolic dysregulation is associated with the various progressions of different cancers, including tumour growth, metastasis, angiogenesis and drug resistance.^5^, ^6^ Recently, several metabolic rate‐limiting enzymes in glycolysis, gluconeogenesis, pentose phosphate pathway (PPP), and fatty acid oxidation (FAO) and tricarboxylic acid (TCA) cycles^7^, ^8^ have been identified as biomarkers and drug targets, such as phosphofructokinase platelet (PFKP),^9^ fructose‐1,6‐bisphosphatase 1 (FBP1)^10^ and UDP‐glucose 6‐dehydrogenase (UGDH).^11^ Therefore, identification of the enzymes from metabolic rate‐limiting enzyme database systematically may provide more prognostic biomarkers and therapeutic targets in HCC.

Several studies have kept tabs on the systematic analysis of the role of metabolism in glioblastoma, gastric cancer and endometrial cancer.^12^, ^13^ However, the comprehensive analysis of the interrelation between rate‐limiting enzymes and clinical prognosis of HCC patients has not been reported yet. In this study, we found that metabolic pathways were more enriched in diethylnitrosamine (DEN) and CCL_4_ induced HCC animal model. Meanwhile, by conjointly analysing the RNA sequencing (RNA‐seq) data of animal model and TCGA data of HCC, we found a series of abnormal metabolic rate‐limiting enzymes involved in HCC progression. Then, we established a signature based on RRM1, UCK2 and G6PD, which was also validated by an independent HCC cohort. Taken together, our study demonstrated abnormal metabolic pathways were involved in HCC development and provides a novel signature based on metabolic rate‐limiting enzyme for predicting the clinical outcome of HCC patients.

## 2 MATERIALS AND METHODS

### 2.1 Mouse models for HCC

The C57BL/6 background mice were purchased from Nanjing Biomedical Research Institute of Nanjing University (Nanjing, Jiangsu, China) and maintained in SPF facilities. At 14 days, the mice were injected with the carcinogen DEN (25 mg/kg, Sigma‐Aldrich), and flowing by intraperitoneal injections of 10% CCl_4_ (5 mg/kg, once a week, Sigma‐Aldrich) at age of 4 weeks. The ultrasonic inspection was performed at indicated time. The mice were sacrificed at 5 months finally. Part of the tumour tissues and normal tissues was fixed in 4%paraformaldehyde for H&E analysis, and the remaining tissues were placed in liquid nitrogen for RNA‐seq, qRT‐PCR and Western blotting.

### 2.2 RNA‐seq analysis

Total RNA was extracted from mouse liver tumour tissues (n = 3) and paired normal tissues (n = 3). The quality and quantity of the RNA were assessed by a NanoDrop TM ND‐1000. Denaturing agarose gel electrophoresis was used to assess RNA integrity. The mRNA extraction was performed using a NEB Next RPoly(A) mRNA Magnetic Isolation Module. RNA libraries were constructed using a KAPA Stranded RNA‐Seq Library Prep Kit (Illumina). Libraries were sequenced using Illumina HiSeq 4000 platforms. The RNA sequencing service was provided by ShuPu (Shanghai, China) BIOTECHNOLOGY LLC. R packages were used to screen the differentially expressed genes between tumour and normal tissues, Gene Ontology (GO) and KEGG pathway analyses were performed to explore the biological functions using ‘clusterprofiler’ R package, and enrichment analysis was presented by R packages ‘ggplot2’ and ‘GO plot’.

### 2.3 Data collection

111 human rate‐limiting metabolic enzymes were selected from the rate‐limiting enzymes database according to previous study,^14^and the complete list of these genes encoding the enzymes is included in Table S1.

All the human RNA expression data and corresponding clinical information of 374 HCC patients were downloaded from TCGA (PanCancer Atlas)(https://tcga‐data.nci.nih.gov/tcga/), and differentially expressed metabolic rate‐limiting enzymes were screened by R package ‘limma’ with a cut‐off criterion of |logFC| > 1 and *P* < .05. The clinical characteristics of enrolled patients from the three HCC cohorts were included in Table S2. The expression level of prognostic associated rate‐limiting metabolic enzymes between cancerous and normal samples was displayed via package ‘pheatmap’ and ‘ggplot’ respectively.

### 2.4 Human specimens

All HCC patients in our independent cohort were included from the Nanjing Drum Tower Hospital, the Affiliated Hospital of Nanjing University Medical School (Nanjing, Jiangsu, China). The cohort included 90 cases including HCC and associated non‐cancerous tissues, which were embedded with paraffin to make the tissue microarray (TMA) and clinicopathological features, which included age, sex, TNM stage (American Joint Committee on Cancer classification, AJCC). Meanwhile, 20 pathologically confirmed HCC and associated non‐cancerous fresh‐frozen tissues from recent patients from the Nanjing Drum Tower Hospital were obtained for qRT‐PCR and Western blotting after informed consent was signed. This study was approved by the Institutional Review Boards of Nanjing Drum Tower Hospital.

### 2.5 RNA extraction and qRT‐PCR analysis

Total RNAs were extracted from human and mouse HCC tissues and associated non‐cancerous tissues using TRIzol reagent (Invitrogen) according to the manufacturer's instructions. The reverse transcription reaction (RT) was performed with Reverse Transcription kit (Vazyme). The RT‐PCR reactions were performed with a SYBR Green PCR Kit (Vazyme), measured in triplicate and performed on an Applied Biosystems 7900HT sequence detection system (Applied Biosystems). GAPDH was used as an internal control for mRNA. The relative expression levels of the RRM1, UCK2 and G6PD were calculated using the comparative 2^−ΔΔCt^ method. The primers and sequences used were listed below:

**Table:** 

Primer	sequence
RRM1‐Forward	GCCGCCAAGAACGAGTCAT
RRM1‐Reverse	AGCAGCCAAAGTATCTAGTTCCA
UCK2‐Forward	GCCCTTCCTTATAGGCGTCAG
UCK2‐Reverse	CTTCTGGCGATAGTCCACCTC
G6PD‐Forward	CGAGGCCGTCACCAAGAAC
G6PD‐Reverse	GTAGTGGTCGATGCGGTAGA
GAPDH‐Forward	GGAGCGAGATCCCTCCAAAAT
GAPDH‐Reverse	GGCTGTTGTCATACTTCTCATGG

### 2.6 Western blotting

The Western blot protocol was performed as previously described.^15^ The antibodies used were as follows: anti‐RRM1 (Protein tech, 1:1000); anti‐UCK2 (Protein tech, 1:1000); anti‐G6PD (Protein tech, 1:1000); anti‐GAPDH (Beyotime, 1:2000). Universal antibody diluent (WB100D) was purchased from New Cell & Molecular Biotech.

### 2.7 Immunohistochemistry

The immunohistochemistry staining of formalin‐fixed, paraffin‐embedded tissue sections was performed following the manufacturer's instructions.^16^ Briefly, formalin‐fixed, paraffin‐embedded tissue sections were deparaffinized with xylene and decreasing concentrations of ethanol. After antigen retrieval and protein blocking steps, tissue sections were incubated with primary antibodies (anti‐RRM1 (Protein tech, 1:100); anti‐UCK2 (Protein tech, 1:250); anti‐G6PD (Protein tech, 1:100), with the horseradish peroxidase‐coupled polymer secondary antibodies. Secondary antibodies were revealed with the liquid DAB Substrate Chromogen System. The immunohistochemical staining results were assigned an index considering both the intensity of staining and the proportion of tumour cell with an unequivocal positive reaction. For UCK2, G6PD and RRM1, a staining index was determined by multiplying the score for staining intensity with the score for positive area.

### 2.8 Cell culture and siRNAs reagents

HepG2, Hep3B, MHCC‐97H, Huh‐7, and HCCLM3 human hepatoma and normal L02 cell lines were obtained from Nanjing KeyGen Biotech Co. Ltd. All cells were cultured with 5% CO2 in Dulbecco's modified Eagle's medium (DMEM, Invitrogen Life Technologies), supplemented with 100 U/ml penicillin, 100 μg/ml streptomycin and 10% foetal bovine serum. RRM1 siRNA, UCK2 siRNA, G6PD siRNA or their respective negative controls were synthesized by Riobio, and sequences of siRNAs were listed below:

**Table:** 

siRNA	sequence
si‐RRM1#1	5′‐UCUUAAUCGCGUAUAAGGCTT‐3′
si‐RRM1#2	5′‐CCCAACAGGAGGACAGCUUTT‐3′
si‐UCK2#1	5′‐GCGGCGAGCCCUUCCUUAUTT‐3′
si‐UCK2#2	5′‐GCCAGAAGCAGGUGGUCAUTT‐3′
si‐G6PD#1	5′‐GCUCUACGAAGAUCUGGAATT‐3′
si‐G6PD#2	5′‐GCAUUGCACAUCAACGGAUTT‐3′

### 2.9 Clonogenic survival assay

Hep3B cells were transfected with indicated siRNAs of RRM1, UCK2 and G6PD for 48h; then, the cells were trypsinized and cultured in 12‐well plates with 500 cells/well for 2 weeks respectively. For scoring colonies, the cells were fixed in 1ml methanol for 15 min and stained with Giemsa for 10 min. and the number of colonies was quantified, and each colony containing cells >50 were counted.

### 2.10 Statistical analysis

Statistical analysis was performed with the R software (version3.6.1). We used chi‐square test for categorical variables and chi‐square or Fisher's exact test for contingency tables. Univariate Cox regression analysis was performed to identify prognostic associated rate‐limiting metabolic enzymes. Package ‘glmnet’ was used to perform LASSO Cox regression model to select optimal weighting coefficients via penalized maximum likelihood and build a prognostic signature. The formula of the risk score for the prediction of HCC patients’ prognosis was as follows: risk score = the sum of the multivariate Cox regression coefficient ratio of each mRNA multiplied by the expression level of each mRNA. For survival analysis, overall survival was defined as the time from first treatment to death for any cause, and Kaplan‐Meier method and log‐rank test were used to detect potential prognostic factors. For clarify relationship of signature, clinicopathological characteristics and prognosis, univariate Cox regression analysis was performed to find out the independent factors correlated with OS, and then these incorporated significantly factors into the multivariate regression model to foresee the comprehensive effect of these factors. AUC was employed to demonstrate the sensitivity and specificity of different variables by risk estimation and AUC at different cut‐off time was used to measure the predictive accuracy. The ‘pROC’ package was used to perform ROC curve and analyse AUC. The ‘survival ROC’ package was used to perform the time‐dependent ROC curve analysis, the ‘rms’ package to consolidate the risk score and clinical characteristics for nomogram construction. All statistical tests were two‐sided, and *P* < .05 was considered to be significant.

## 3 RESULTS

### 3.1 Abnormal expression of metabolic rate‐limiting enzymes in HCC

**FIGURE 1:**
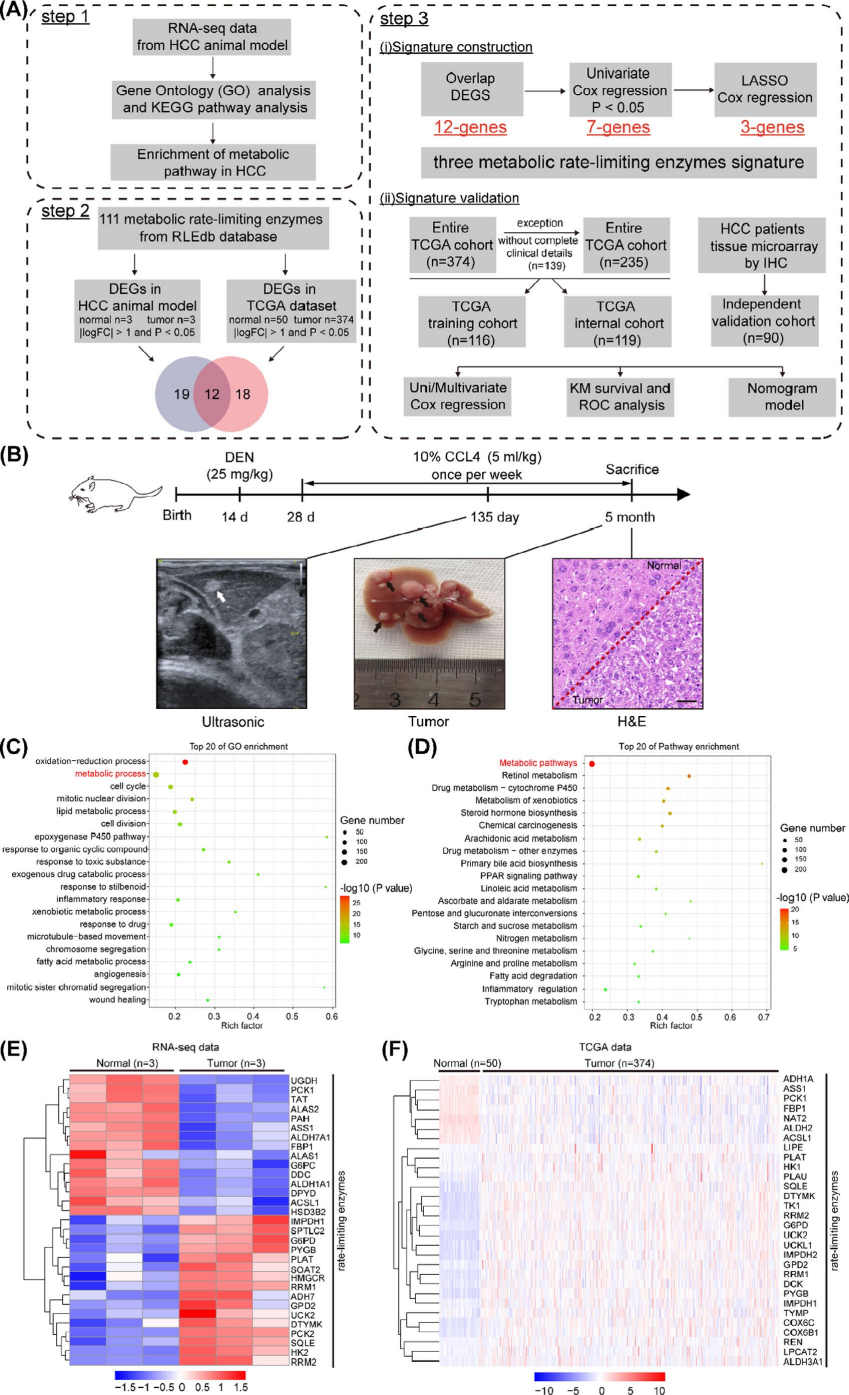
Metabolic rate‐limiting enzymes were differentially expression in HCC. (A) Flow chart of this study. (B) Schematic diagram of DEN+CCL_4_ induced HCC animal model. (C) GO enrichment analysis of differentially expressed rate‐limiting enzymes between mouse liver cancer and paired normal tissues. D, KEGG pathway enrichment analysis of differentially expressed rate‐limiting enzymes between mouse liver cancer and paired normal tissues. (E) Heat map of differentially expressed rate‐limiting enzymes in between mouse liver cancer and paired normal tissues(n = 3). (F) Heat map of differentially expressed rate‐limiting enzymes between TCGA HCC tumour (n = 374) and normal tissues(n = 50)

The detailed flow chart of the study design is displayed in Figure 1A. To investigate the functional pathways in hepatocellular carcinogenesis, we first constructed DEN and CCL_4_ induced HCC mice model. At 135 days, ultrasonic inspection indicated the formation of HCC, and the morphological and H&E staining confirmed the formation of HCC at 5 months (Figure 1B). The mouse HCC tissues and paired normal tissues (n = 3) were then analysed by RNA‐seq. Gene Ontology (GO) and Kyoto Encyclopedia of Genes and Genomes (KEGG) pathway analyses of differentially expressed genes from RNA‐seq data clustered the majority genes in metabolic pathways, indicating metabolic pathways played a key role in the occurrence and development of HCC (Figure 1C,D). The metabolic pathways included lipid, fatty acid metabolic progress, retinol metabolism and PAR signalling pathway (Figure 1D). Furthermore, we compared the transcriptome profiling of 111 rate‐limiting enzymes between the tumour and matched normal tissues from a database of rate‐limiting enzymes reported previously^14^ and found that 31 transcripts were significantly differentially expressed, including 16 up‐regulated and 15 down‐regulated transcripts (Figure 1E).

In addition, we systematically screened the 111 metabolic rate‐limiting enzymes using TCGA databases (including 374 HCC samples and 50 normal samples) and identified 30 differentially expressed transcripts (*P* < .05). Among them, 23 genes were up‐regulated and 7 were down‐regulated in HCC patients (Figure 1F and Figure S1A). GO and KEGG pathway analyses also showed enrichment in genes involved in multiple metabolic processes (Figure S2A–C and Figure S3A–C). These results accordingly indicated metabolic rate‐limiting enzymes and relative pathways were enriched in the development of HCC.

### 3.2 Identification of clinical prognosis of the metabolic rate‐limiting enzymes

**FIGURE 2:**
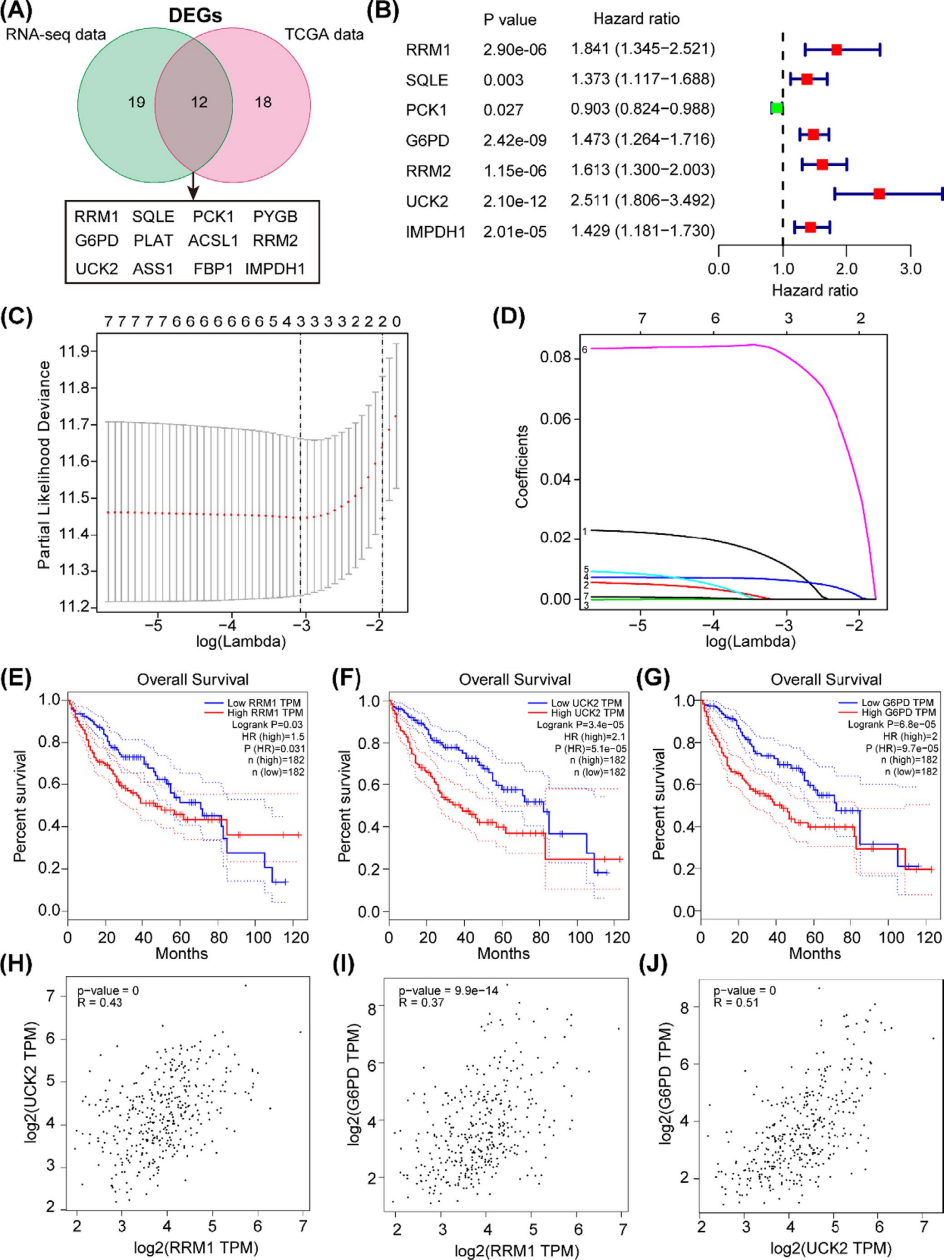
Selection of metabolic rate‐limiting enzymes related to clinical prognosis. (A) RNA‐seq and TCGA data identified differentially expressed rate‐limiting enzymes. (B) Univariate Cox analysis identified seven rate‐limiting enzymes associated with HCC patient's prognosis. (C) Plots of the cross‐validation error rates. Each dot represents a lambda value along with error bars to give a confidence interval for the cross‐validated error rate. (D) LASSO coefficient profiles of the rate‐limiting enzymes associated with the overall survival of HCC. (E‐G) Kaplan‐Meier survival curve of RRM1, UCK2 and G6PD in TCGA HCC data were performed. (H‐J) Spearman's correlation among the RRM1, UCK2 and G6PD in TCGA HCC data was performed (http://gepia.cancer‐pku.cn/)

Intriguingly, by conjointly analysing the differentially expressed metabolic rate‐limiting enzymes from RNA‐seq data of HCC animal model and TCGA data, 12 transcripts were overlapped (RRM1, SQLE, PCK1, PYGB, G6PD, PLAT, ACSL1, RRM2, UCK2, ASS1, FBP1 and IMPDH1) (Figure 2A). To better understand the correlation between these overlapped genes and prognostic significance of HCC patients, we utilized univariate Cox regression analysis to screen out 7 rate‐limiting enzymes with a significant overall prognosis (RRM1, SQLE, PCK1, G6PD, RRM2, UCK2 and IMPDH1) (Figure 2B). Furthermore, the LASSO Cox regression analysis was used to dissect their effect on clinical prognosis. The regularization path was computed for the LASSO at a grid of values for the regularization parameter lambda, three genes (RRM1, G6PD and UCK2) were sorted out from seven genes and optimal weighting coefficients were selected (Figure 2C,D). As shown in Figure 2E‐G, high expression of RRM1 (*P* = .03), UCK2 (*P* = 3.4e‐05) and G6PD (*P* = 6.8e‐05) transcript in HCC tissues from TCGA was significantly associated with poor overall survival (OS) respectively. We also analysed the correlation among the RRM1, UCK2 and G6PD using TCGA data and found that there was significant correlation between RRM1, UCK2 and G6PD (RRM1 *vs* UCK2: R = 0.43; RRM1 *vs* G6PD: R = 0.37; UCK2 *vs* G6PD: R = 0.51) (Figure 2H‐J).

### 3.3 RRM1, UCK2 and G6PD are highly expressed in HCC and are associated with overall survival

**FIGURE 3:**
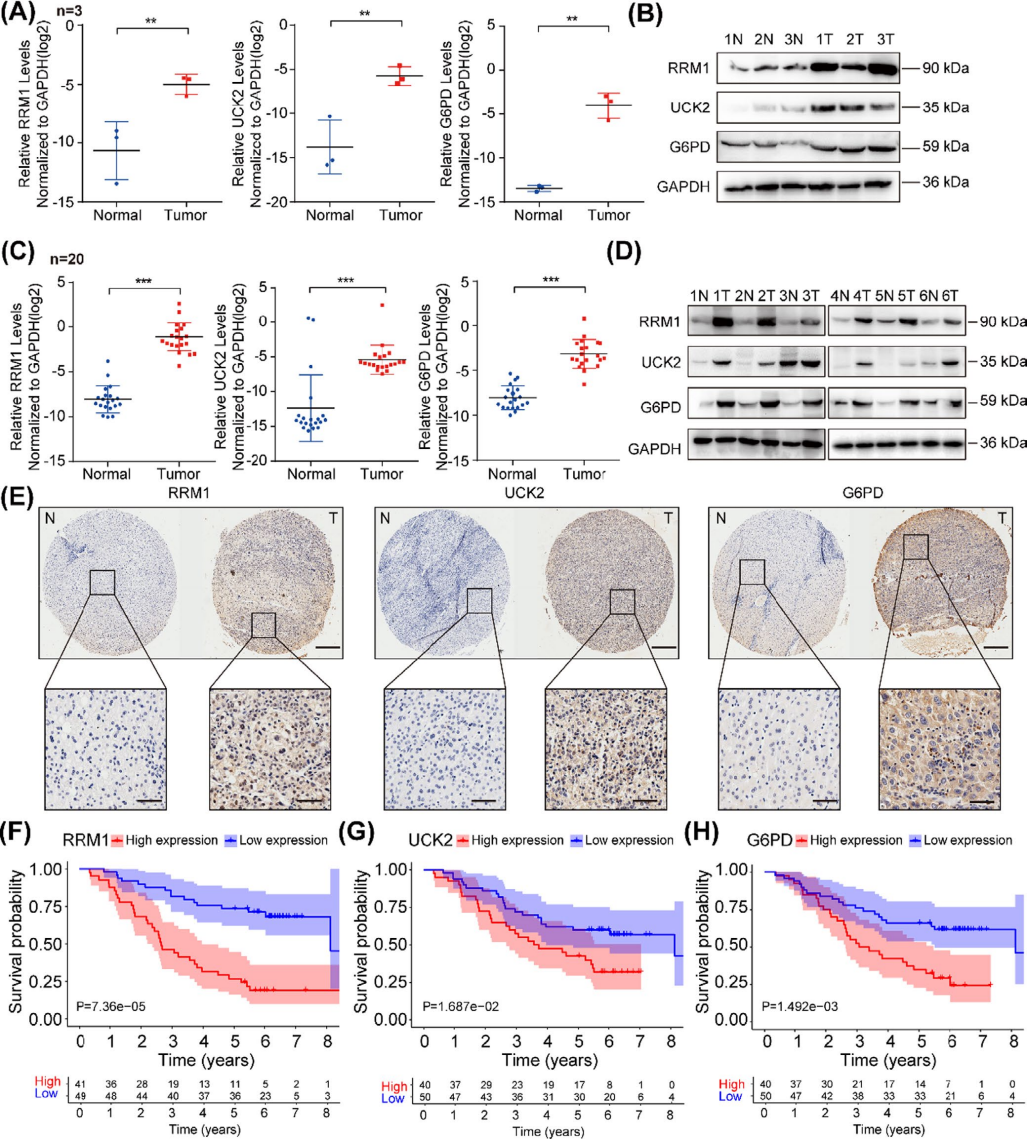
Verification the expression and clinical prognosis of RRM1, UCK2 and G6PD in HCC. (A‐B) The expression level of RRM1, UCK2 and G6PD in mouse liver cancer and paired normal tissues was determined by qRT‐PCR and Western blotting (n = 3). (C) The expression level of RRM1, UCK2 and G6PD in cancerous tissues and corresponding normal tissues from HCC patients was determined by qRT‐PCR (n = 20). (D) Western blotting was applied to determine the expression of RRM1, UCK2 and G6PD in cancerous tissues and corresponding normal tissues (n = 6). The data are the mean ± SD of three independent experiments. (E) Representative IHC images from HCC cohort probed with the anti‐RRM1, UCK2 and G6PD antibody (scale bars: 50 or 500 μm respectively) were shown. (F‐H) Kaplan‐Meier survival curve of RRM1, UCK2 and G6PD was performed in the HCC cohort. The data are the mean ± SD of three independent experiments. **P < .01; ***P < .001

To detect the expression of RRM1, UCK2 and G6PD in RNA‐seq data from HCC animal model, we first detect their expression in the mouse liver cancer tissues and paired normal tissues, and the results showed that the mRNA and protein levels of RRM1, UCK2 and G6PD were significantly higher in tumour tissues compared with those in normal tissues by qRT‐PCR and Western blotting assays (Figure 3A‐B). Consistently, the high levels of them were also confirmed in 20 human HCC tissues compared to the corresponding normal tissues (Figure 3C‐D).

Considering the rate‐limiting enzyme performs important biological function based on its protein levels, we then utilized a blinded external independent validation cohort (n = 90) to further investigate the RRM1, UCK2 and G6PD expression and their relationship with clinical outcome using immunohistochemistry (IHC) staining in HCC TMA. Similarly, the results showed that the protein levels of RRM1, UCK2 and G6PD were significantly increased in HCC tissues compared to those in matched normal tissues (n = 90, *P* < .01; Figure 3E and Figure S4A). Simultaneously, univariate Cox regression analysis demonstrated that the protein levels of RRM1, UCK2 and G6PD were significantly correlated with OS in HCC patients (Figure S4B). Furthermore, multivariate Cox regression analysis also revealed that RRM1, UCK2 and G6PD could act as an independent predictive marker for the prognosis of HCC (Figure S4C). Meanwhile, Kaplan‐Meier survival analyses showed that HCC patients with high levels of RRM1, UCK2 and G6PD had worse OS (*P* = 7.36e‐05; *P* = 1.687e‐02; *P* = 1.492e‐03 respectively) (Figure 3F‐H). Taken together, these results suggest that the expression of RRM1, UCK2 and G6PD are up‐regulated in HCC and all of them could act as an independent prognostic factor for HCC.

### 3.4 Construction of a signature based on the metabolic rate‐limiting enzymes

**FIGURE 4:**
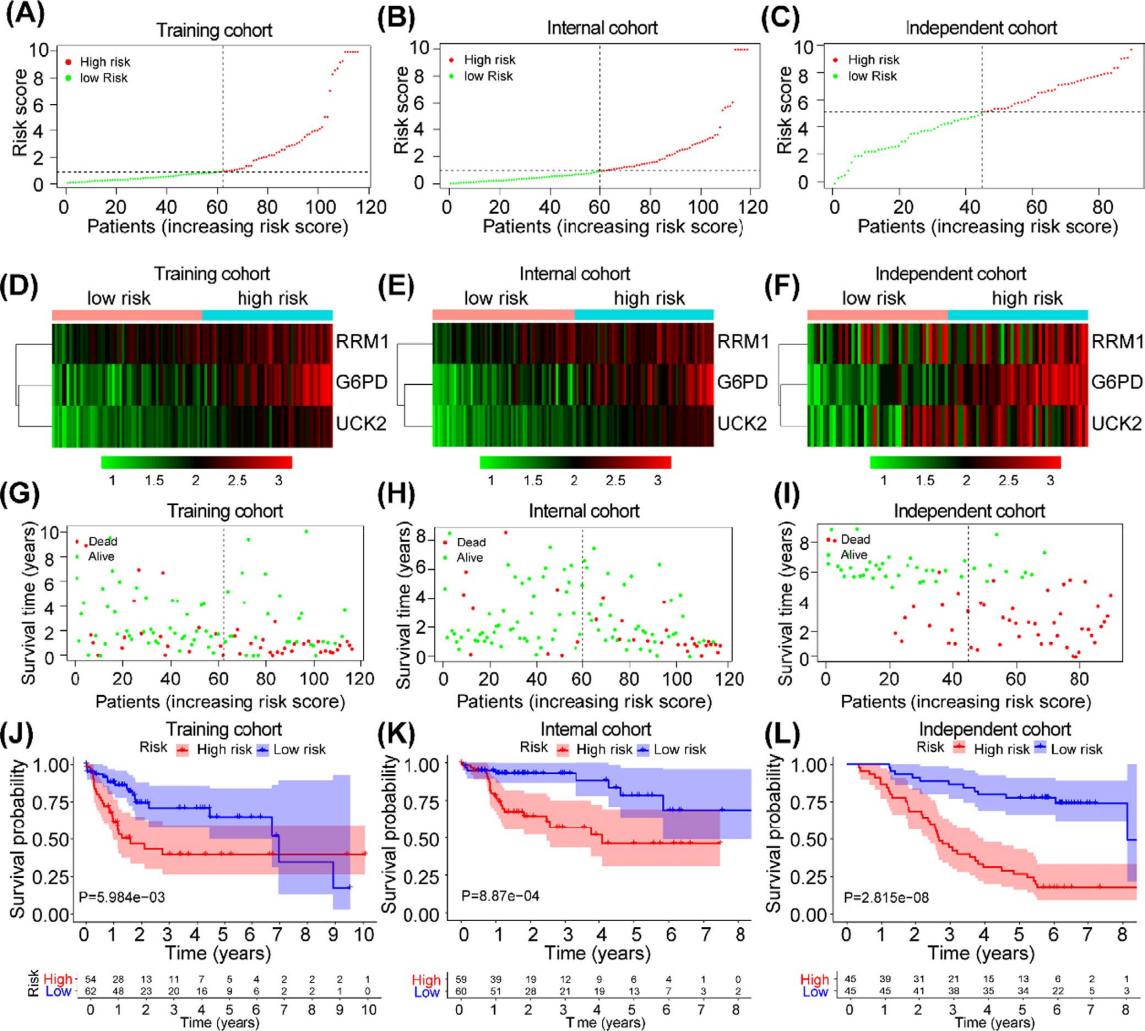
Construction and Validation of a metabolic rate‐limiting enzymes signature. Validation of the efficacy of the risk signature in three cohorts. (A‐D) The distribution, heat maps, survival status and Kaplan‐Meier survival curve in the TCGA testing cohort. (E‐H) The distribution, heat maps, survival status and Kaplan‐Meier survival curve in the TCGA internal cohort. (I‐L) The distribution, heat maps, survival status and Kaplan‐Meier survival curve in the independent cohort

Based on the expression levels of RRM1, UCK2 and G6PD from entire TCGA data cohort, the following formula was derived to calculate prediction model risk score for each patient: Risk score = (0.006 × expression value of RRM1) + (0.075 × expression value of UCK2) + (0.005 × expression value of G6PD). Then, we randomly divided the 235 HCC patients from TCGA into the training cohort (n = 116) and the internal cohort (n = 119). With the risk score formula, patients in the training cohort were stratified into high‐risk (n = 54) and low‐risk (n = 62) subgroups according to mean risk score (Figure 4A), and the expression levels of RRM1, UCK2 and G6PD were higher in the high‐risk group (Figure 4D). Furthermore, it showed that the patients in high‐risk group had shorter OS than those in the low‐risk group (Figure 4G, J).

To further validate the efficiency of this signature, the internal cohort (n = 119) and the total HCC patients from TCGA data (n = 235) were used to assess (Figure 4B and Figure S5A), as shown in Figure 4E and Figure S5B, the expression of these three genes was higher in the high‐risk subgroup. Survival analysis of the two cohorts also showed that HCC patients in the high‐risk group had worse prognosis than those in the low‐risk group (Figure 4H, K and Figure S5C, D), which further confirmed the results in the training cohort.

We then further assess the predictive ability of the signature in external independent validation cohort (Figure 4C). Intriguingly, it showed that HCC patients with high‐risk scores generally had higher protein levels of RRM1, UCK2 and G6PD than those with low scores (Figure 4F), and HCC patients in the low‐risk group had a better prognosis than those in the high‐risk group (Figure 4I, L).

### 3.5 The prognostic value of the signature in three cohorts of HCC

**FIGURE 5:**
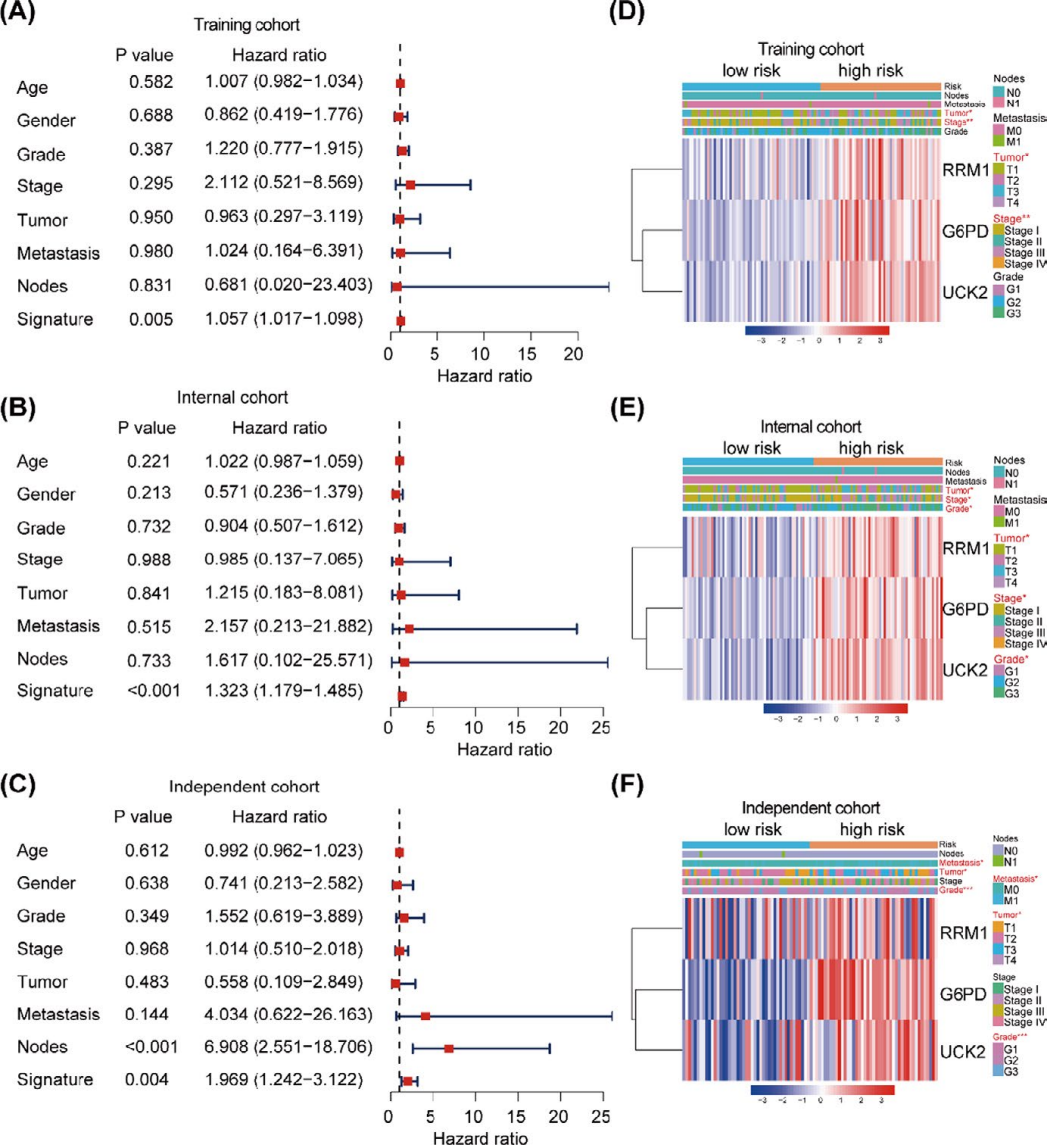
Multivariate Cox analysis in three HCC cohorts. (A‐C) Multivariate Cox analysis was performed in the TCGA testing cohort, TCGA internal cohort and independent cohort. (D‐F) Heat maps of the expression of RRM1, UCK2 and G6PD with corresponding clinicopathological characteristics in the TCGA testing cohort, TCGA internal cohort and independent cohort. **P < .01; ***P < .001

To further clarify the relationship between the signature and clinicopathological characteristics and prognosis in HCC patients, we performed univariate and multivariate Cox regression analysis. Univariate Cox regression analyses indicated that TNM stage (HR = 1.865, 95% CI: 1.456‐2.388) and our signature (HR = 2.794, 95% CI: 1.991‐3.920) were independent risk factors for OS in the entire TCGA cohort, similar with that of a published 10‐gene metabolic signature (Weng_ signature, HR = 3.203, 95% CI: 2.383‐4.304).^17^Furthermore, our signature had a relatively better predictive output than a 4‐gene metabolic signature (Liu_ signature, HR = 1.597, 95% CI: 1.125‐2.267) (Figure S5E).^18^Subsequent multivariate Cox regression analyses also revealed that the signature was an independent risk factor for poor OS in HCC patients (Our signature, HR = 2.512, 95% CI: 1.754‐3.598; Weng_ signature, HR = 3.194, 95% CI: 2.271‐4.492; Liu_signature, HR = 1.510, 95% CI: 1.040‐2.193) (Figure S5F). And in the training cohort and internal cohort, univariate Cox regression analysis showed that TNM stage, tumour burden and signature were significantly correlated with OS in HCC patients (Figure S6A,B), while multivariate Cox regression analysis showed only the signature had an independent prognostic ability (Figure 5A,B). In the external independent validation cohort, univariate Cox regression analysis also showed that TNM stage, differentiation grade, tumour burden, lymph node metastasis and signature were substantially associated with survival in HCC patients (Figure S6C), while multivariate Cox regression analysis showed that the signature and lymph node metastasis were indicated as independent predictive marker for the prognosis of HCC patients (Figure 5C). Next, we compared our classifier with the existing clinicopathological features, it showed that, in the training cohort, TNM stage and tumour burden were significantly associated with risk score (Figure 5D), which were confirmed in the internal cohort (Figure 5E). Consistently, in the independent validation cohort, more patients with advanced TNM stage, serous tumour burden and high grade were seen in high‐risk group compared with low‐risk group (Figure 5F).

**FIGURE 6:**
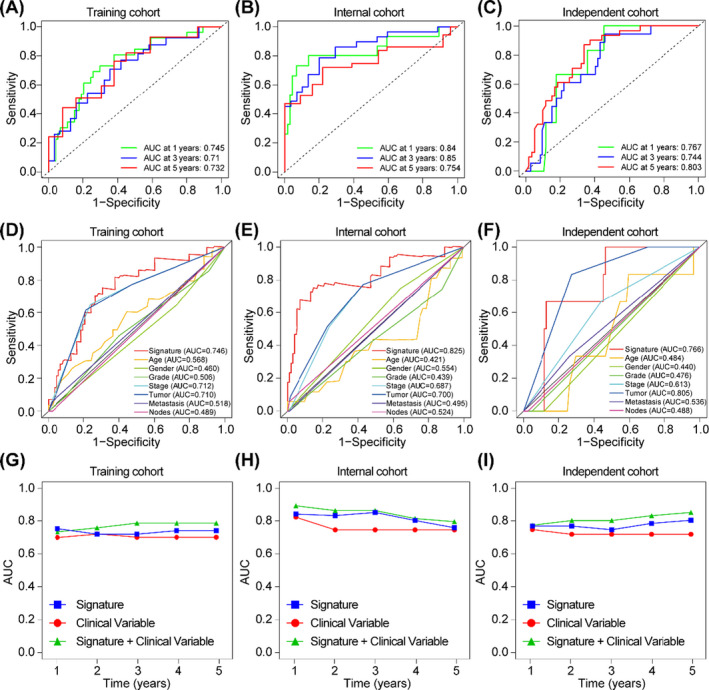
ROC curves and AUC values. (A‐C) Time‐dependent ROC curve in the TCGA testing cohort, TCGA internal cohort and independent cohort to assess prognostic accuracy. (D‐F) Comparisons of the prognostic accuracy for signature and clinicopathological characteristics (Age, gender, grade, stage, tumour, metastasis, lymph node metastasis) in the TCGA testing cohort, TCGA internal cohort and independent cohort. (G‐I) Time‐dependent ROC curve for signature, clinical variable (TNM stage and grade) and the combined signature and clinical variable in the TCGA testing cohort, TCGA internal cohort and independent cohort

To further assess the accumulative effects of the metabolic rate‐limiting enzymes signature on the prediction of OS, we calculated the area under the receiver operating characteristic curve (AUC) in the training, internal and independent cohorts respectively. Time‐dependent receiver operating characteristic (ROC) analysis showed that the AUC at 1‐, 3‐ and 5‐year OS of the signature were 0.745, 0.71 and 0.732 in training cohort (Figure 6A), 0.84, 0.85 and 0.754 in internal cohort (Figure 6B), and 0.767, 0.744 and 0.803 in independent cohort, respectively (Figure 6C), indicating that this signature is a stable predictor along with censored survival data. Next, we compared the prognostic efficacy of the signature with each of the single clinicopathological risk factor, and the results showed that AUC of the signature was significantly higher than any other single clinicopathological risk factors (AUC of signature was 0.746 in training cohort, 0.825 in internal cohort, and 0.766 in independent cohort respectively) (Figure 6D‐F). To improve the predictive efficacy, the risk scores of the signature and clinical variable (TNM stage and grade) were combined. It was shown that combination of the clinical risk score (TNM stage, histologic type and tumour diameter) and the signature contributed much more than either one alone in all of three cohorts. For example, in the independent cohort, the AUC at year 5 was 0.715 (95% CI: 0.653‐0.777) for clinical risk score, whereas it was significantly increased to 0.852 (95% CI: 0.798‐0.906) when combination of the clinical risk score with the signature risk score (Figure 6G‐I).

### 3.6 Development of a prognostic nomogram to predict the individual outcomes of HCC patients

Based on the signature and clinicopathological characteristics, we built a comprehensive prognostic nomogram to estimate overall survival probability for 5 years in HCC patients using the independent cohort. Two independent prognostic parameters (metabolic rate‐limiting enzymes signature and lymph node metastasis) were integrated into the nomogram, which showed that higher total score was associated with shorter OS of HCC patients (Figure S6D). Furthermore, we found the predicted value was more consistent with the actual value, which were confirmed by the calibration curve of the nomogram for the survival probability at 3 or 5 years (Figure S6E,F).

### 3.7 RRM1, UCK2 and G6PD promote HCC proliferation in vitro

**FIGURE 7:**
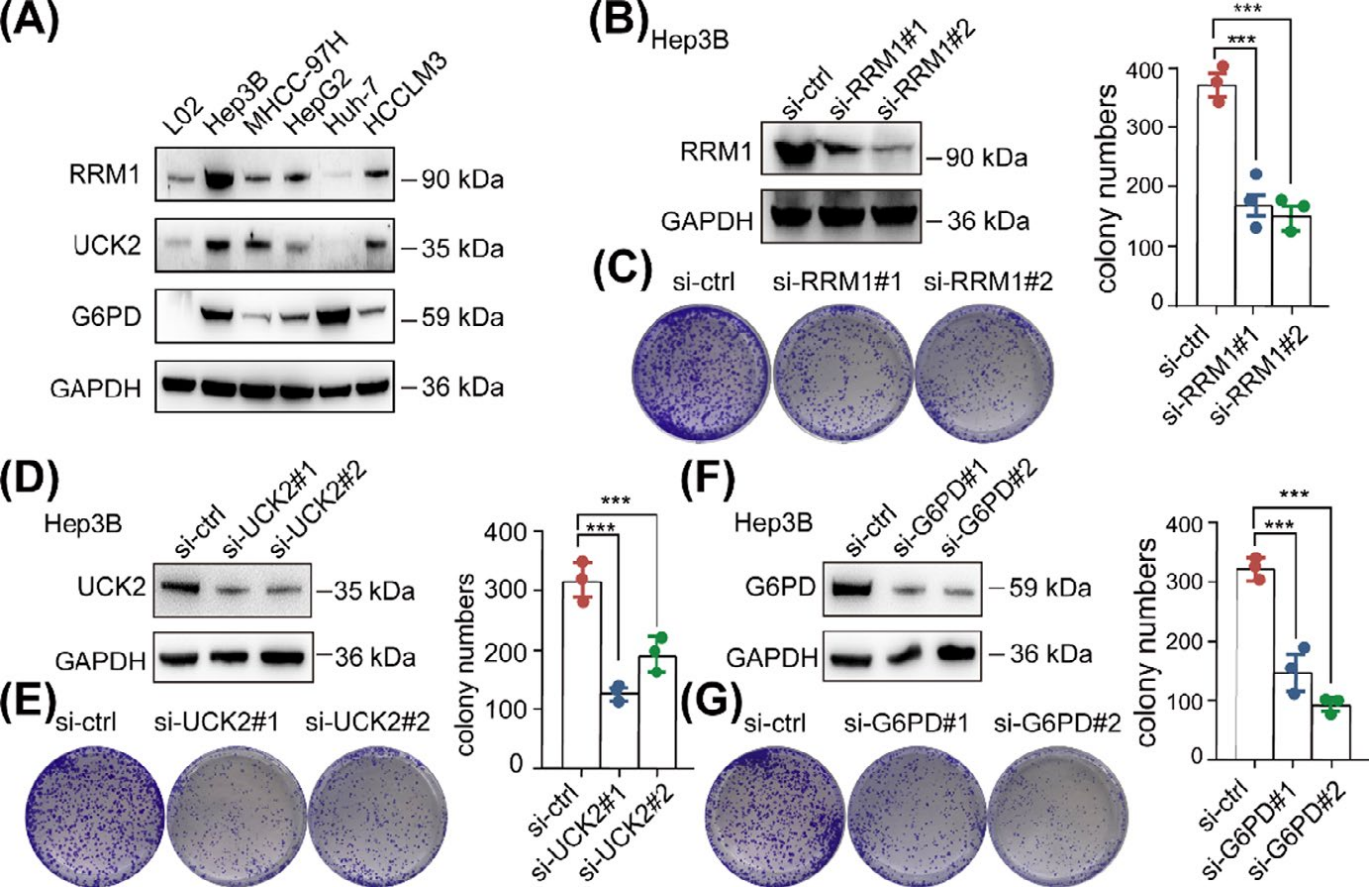
RRM1, UCK2 and G6PD promotes HCC proliferation in vitro. (A) Western blotting analysis of RRM1, UCK2 and G6PD expression level in normal hepatocytes and five HCC cell lines (Hep3B, MHCC‐97H, HepG3, Huh‐7 and HCCLM3). (B) The protein level of RRM1 in Hep3B cells with two specific siRNAs targeting RRM1 was measured by Western blotting. (C) The clonogenic ability in Hep3B cells with RRM1 two specific siRNAs was determined (left). Quantification of the colony formation assay results (right). (D) The protein levels of UCK2 in Hep3B cells with two specific siRNAs targeting UCK2 were measured by Western blotting. (E) The clonogenic ability in Hep3B cells with UCK2 two specific siRNAs was determined (left). Quantification of the colony formation assay results (right). (F) The protein levels of G6PD in Hep3B cells with two specific siRNAs targeting G6PD were measured by Western blotting. (G) The clonogenic ability in Hep3B cells with G6PD two specific siRNAs was determined (left). Quantification of the colony formation assay results (right). The data are the mean ± SD of three independent experiments. **P < .01; ***P < .001

To further elucidate the function of these three rate‐limiting enzymes in HCC cells, we first investigated RRM1, UCK2 and G6PD expression levels in HCC cell lines and normal hepatocytes by Western blot analysis. Our data suggested that RRM1 and UCK2 were preferentially expressed in most HCC cell lines, except for Huh7 cell, whereas high expression of G6PD was observed in Hep3B and Huh7 cells (Figure 7A). Subsequently, we transfected Hep3B cell with two specific siRNAs of RRM1, UCK2 and G6PD, and the protein level of RRM1, UCK2 and G6PD was markedly reduced respectively (Figure 7B,D,F). We also performed colony formation studies to elucidate the functional roles of the three in vitro, and the results showed that knockdown of RRM1, UCK2 and G6PD significantly suppressed the clonogenic ability of HCC cells (Figure 7C,E,G). Taken together, these data suggested that RRM1, UCK2 and G6PD may act as oncogene that promotes HCC proliferation.

## 4 DISCUSSION

Recently, accumulating investigations have demonstrated that metabolic dysregulation plays a critical role in the malignant process of various cancers.^19^, ^20^ Metabolic rate‐limiting enzymes are the executive medium of energy metabolism to drive the occurrence and development of cancer. For instance, Polo‐like kinase 3 (PLK3) could inhibit glucose metabolism by targeting HSP90/STAT3/HK2 signalling, indicating it may serve as a potential therapeutic target in colorectal cancer.^21^ Sterol O‐acyltransferase 1 (SOAT1) is a key enzyme which could convert endoplasmic reticulum (ER) cholesterol to cholesterol esters (CE) to store in lipid droplets (LDs), and inhibition of SOAT1 could effectively suppress SREBP‐1 and block glioblastoma growth.^22^ In our study, we found RRM1, UCK2 and G6PD are significantly increased in HCC and closely associated with poor prognosis of HCC patients.

RRM1 could regulate metabolization and activity of mitotane.^23^ Previous research has demonstrated that higher RRM1 expression is discovered to associated with shorter overall survival in non‐small cell lung cancer (NSCLC),^24^ non‐muscle‐invasive bladder cancer (NMIBC)^25^ and multiple myeloma patients.^26^ UCK2 could convert uridine and cytidine to uridine monophosphate (UMP) and cytidine monophosphate (CMP), promoting metastasis of HCC cells via the STAT3/MMP2/MMP9 signal axis.^27^ UCK2 has also been recognized as an indicator of unfavourable prognosis in HCC and breast cancer, which was similar to our result.^28^, ^29^ In addition, G6PD is the production of ribose and the reducing equivalent nicotinamide adenine dinucleotide phosphate (NADPH) via the PPP. G6PD could enhance tumour growth by maintaining intracellular redox homeostasis.^30^ G6PD activity is increased in several types of cancers, including bladder cancer, breast cancer, prostate cancer and ovarian cancer.^31^, ^32^, ^33^ In our study, we preliminary reveal knockdown of RRM1, UCK2 and G6PD significantly suppressed the clonogenic ability of HCC cells, which suggests that these three rate‐limiting enzymes may act as oncogene that promotes HCC proliferation.

However, increasing studies have indicated that single clinical factor or single gene feature are susceptible to the multiple factors, which makes it difficult to be true and reliable prognostic marker.^34^ With the swift development of high‐throughput sequencing technology, it may be conceivable to focus on systematic exploration of a class of genes associated with prediction of patient’ survival.^35^ For instance, Weng, et al^17^ developed a prognostic signature based on 10 metabolic genes, which reflects the metabolic and immune characteristics of tumours. Another study has identified a novel robust four‐gene metabolic signature to explain the dysregulated metabolic microenvironment and pathways.^18^In our study, by comparing the differentially expressed metabolic rate‐limiting enzymes from the RNA‐seq of animal model and TCGA data in HCC, 12 transcripts were overlapped. Meanwhile, univariate Cox regression analysis screened out seven rate‐limiting enzymes with a significant overall prognosis of HCC patients. Furthermore, LASSO Cox regression was performed to identify a combination of three‐metabolic rate‐limiting enzyme signature (RRM1, UCK2 and G6PD) for prediction of the prognosis of HCC patients. Subsequent survival analysis also showed that high‐risk score group was related to poorer prognosis in three independent cohorts. In addition, metabolic rate‐limiting enzyme signature and clinical characteristics were identified as independent overall survival‐related variables, which were incorporated into the nomogram, indicating that the nomogram can be regarded as a valid tool for clinical diagnosis and treatment of HCC patients. Furthermore, we utilized univariate and multivariate Cox regression analyses to compare our signature with the published signatures, and the results indicated that our signature similar with that of a published 10‐gene metabolic signature (Weng_ signature) and had a relatively better predictive output than the 4‐gene metabolic signature. These findings suggested that this signature had powerful capacity to predict the prognosis of HCC patients, which may be helpful to guide significance for the decision‐making of clinical treatment.

In summary, for the first time, we identified and validated a novel signature based on three‐metabolic rate‐limiting enzymes, which was associated with the prognosis of HCC patients. Our study not only demonstrates abnormal metabolic pathways contribute to HCC progression, but more importantly provides a new metabolic rate‐limiting enzyme prognostic signature for HCC, which was useful for accurate risk assessment in guiding clinical management and treatment decisions.

## CONFLICT OF INTEREST

The authors declare no competing interests.

## AUTHOR CONTRIBUTIONS

ZW, XZ, QW and SW designed the project. ZW and CC collected and analysed the TCGA data and RNA‐seq data. ZW and YF evaluated the IHC of TMA. AX and GX provided clinical samples and collected clinical information. ZW and CC performed the experiments. ZW drafted the manuscript. QW and SW revised this manuscript. All authors read and approved the final manuscript.

## Supporting information

Supplementary Material

Fig S1‐S6

## Data Availability

The data used to support the findings of this study are from public database, and the website has been provided in the corresponding position of the manuscript. The experiment data are available upon reasonable request.
