# Supplementary material for: Prognostic and predictive role of a metabolic rate‐limiting enzyme signature in hepatocellular carcinoma
Source: Cell Prolif. 2021 Aug 23;54(10):e13117. doi: 10.1111/cpr.13117 (PMC8488553; doi:10.1111/cpr.13117)
Supplement: Supplementary file 1 — Supplementary Material [file CPR-54-e13117-s001.docx]

**Supplementary figures and tables：**

**Figure S1 Metabolic rate-limiting enzymes were differentially expression in TCGA HCC data.** A, Boxplot of differentially expressed rate-limiting enzymes between TCGA HCC tumor (n=374) and normal tissues(n=50).

**Figure S2 GO enrichment analysis of differentially expressed rate-limiting enzymes.** A, GO analysis showed the biological processes, cellular component, and molecular functions involved in differential genes, the node color changes gradually from red to blue in descending order according to the adjusted P-values. The size of the node represents the number of counts. B, Circle plot of the enriched rate-limiting enzymes in the enriched GO items. C, Heatmap of the expression of rate-limiting enzymes in the enriched GO items.

**Figure S3 KEGG pathway enrichment analysis of differentially expressed rate-limiting enzymes.** A, KEGG analysis showed significantly enriched pathways of rate-limiting enzymes. The node color changes gradually from red to blue in descending order according to the adjusted P-values. The size of the node represents the number of counts. B, Circle plot of the enriched rate-limiting enzymes in the KEGG items. C, Heatmap of the expression of rate-limiting enzymes in the enriched KEGG items.

**Figure S4 Verification the clinical prognosis of RRM1, UCK2, and G6PD in HCC.** A, The distribution of the difference in RRM1(left), UCK2(middle), and G6PD(right) immunoreactivity score (IRS) (△IRS=IRS of Tumor − IRS of Normal) (n=90 *P*<0.01). B, Univariate Cox analysis was performed in the HCC cohort. C, Multivariate Cox analysis was performed in the HCC cohort. ***P*< 0.01; ****P*< 0.001.

**Figure S5 Estimation of the efficacy of the signature in TCGA entire HCC cohort.** A, The distribution of risk score. B, Expression heatmaps of RRM1, UCK2, and G6PD. C, survival status in TCGA entire HCC cohort. D, Kaplan-Meier survival curve in TCGA entire HCC cohort. E-F, Univariate Cox analysis and Multivariate Cox analysis in TCGA entire HCC cohort and the published signatures.

**Figure S6 Nomogram for predicting survival probability of patients with HCC.** A-C, Univariate Cox analyses were performed in the TCGA testing cohort, TCGA internal cohort, and independent cohort. D, Prognostic nomogram for HCC patients. E-F, Calibration curves for the nomogram at 3- and 5-year.

**Supplementary Table 1**

**The list of genes encoding 111 rate-limiting metabolic enzymes**

| ACO1 | ALDH7A1 | DPYD | HK2 | NAT1 | PYGM | TK1 |
| --- | --- | --- | --- | --- | --- | --- |
| ACO2 | ALDH9A1 | DTYMK | HK3 | NAT2 | RDH5 | TPH1 |
| ACSL1 | ALOX5 | F2 | HMGCR | OGDH | REN | TPH2 |
| ADH1A | APRT | FBP1 | HMGCS1 | OGDHL | RRM1 | TYMP |
| ADH7 | ASS1 | FBP2 | HMGCS2 | PAH | RRM2 | TYR |
| ADK | BACE1 | G6PC | HSD17B6 | PCK1 | RRM2B | UCK1 |
| ALAD | CHAT | G6PC2 | HSD3B1 | PCK2 | SAT1 | UCK2 |
| ALAS1 | COX4I1 | G6PD | HSD3B2 | PIK3C3 | SCD | UCKL1 |
| ALAS2 | COX5B | GAD1 | IMPDH1 | PKLR | SCD5 | UGDH |
| ALDH1A1 | COX6A1 | GAD2 | IMPDH2 | PKM2 | SOAT1 | UGT2B4 |
| ALDH1A2 | COX6A2 | GGT1 | LPCAT2 | PLAT | SOAT2 | XDH |
| ALDH1A3 | COX6B1 | GNE | LTA4H | PLAU | SPTLC1 |  |
| ALDH1B1 | COX6C | GPAM | LTC4S | PTGS1 | SPTLC2 |  |
| ALDH2 | DDC | GPD2 | MYLK | PTGS2 | SQLE |  |
| ALDH3A1 | DLD | HDC | MYLK2 | PYGB | STS |  |
| ALDH3A2 | DLST | HK1 | MYLK3 | PYGL | TAT |  |

**Supplementary Table 2**

**Clinical characteristics of enrolled patients from the three HCC cohorts**

|  | TCGA training cohort |  | TCGA internal cohort |  | External independently cohort |
| --- | --- | --- | --- | --- | --- |
|  | N=116 |  | N=119 |  | N=90 |
| Age |  |  |  |  |  |
| ≥60 | 34 |  | 46 |  | 14 |
| <60 | 82 |  | 73 |  | 76 |
| Gender |  |  |  |  |  |
| Male | 41 |  | 33 |  | 80 |
| Female | 75 |  | 86 |  | 10 |
| Differentiation grade |  |  |  |  |  |
| Well | 20 |  | 9 |  | 15 |
| Moderate | 47 |  | 55 |  | 30 |
| Poor | 49 |  | 55 |  | 45 |
| Tumor |  |  |  |  |  |
| T1 | 43 |  | 71 |  | 24 |
| T2, T3, T4 | 73 |  | 48 |  | 66 |
| Lymph node metastasis |  |  |  |  |  |
| N0 | 109 |  | 115 |  | 88 |
| N1, N2, N3 | 7 |  | 4 |  | 2 |
| Metastasis |  |  |  |  |  |
| M0 | 105 |  | 110 |  | 66 |
| M1 | 11 |  | 9 |  | 24 |
| TNM stage |  |  |  |  |  |
| Ⅰ | 65 |  | 70 |  | 21 |
| Ⅱ-Ⅳ | 51 |  | 49 |  | 69 |
