# Supplementary figures and images for: Prognostic and predictive role of a metabolic rate‐limiting enzyme signature in hepatocellular carcinoma
Source: Cell Prolif. 2021 Aug 23;54(10):e13117. doi: 10.1111/cpr.13117 (PMC8488553; doi:10.1111/cpr.13117)

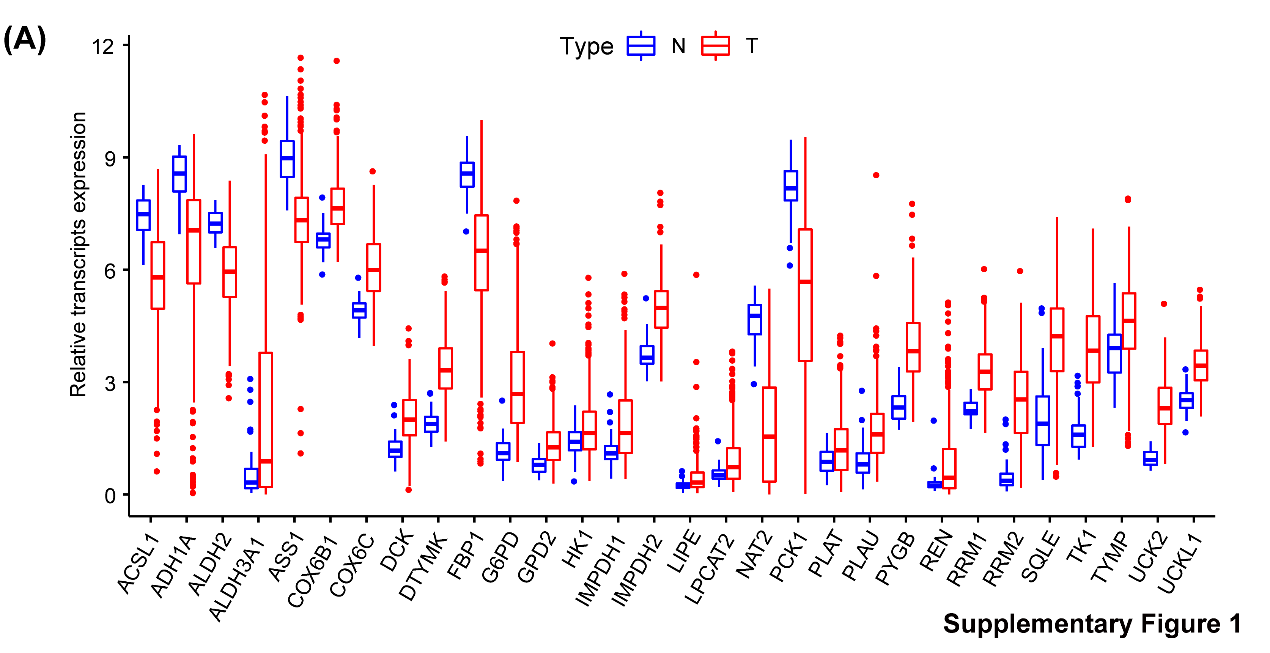


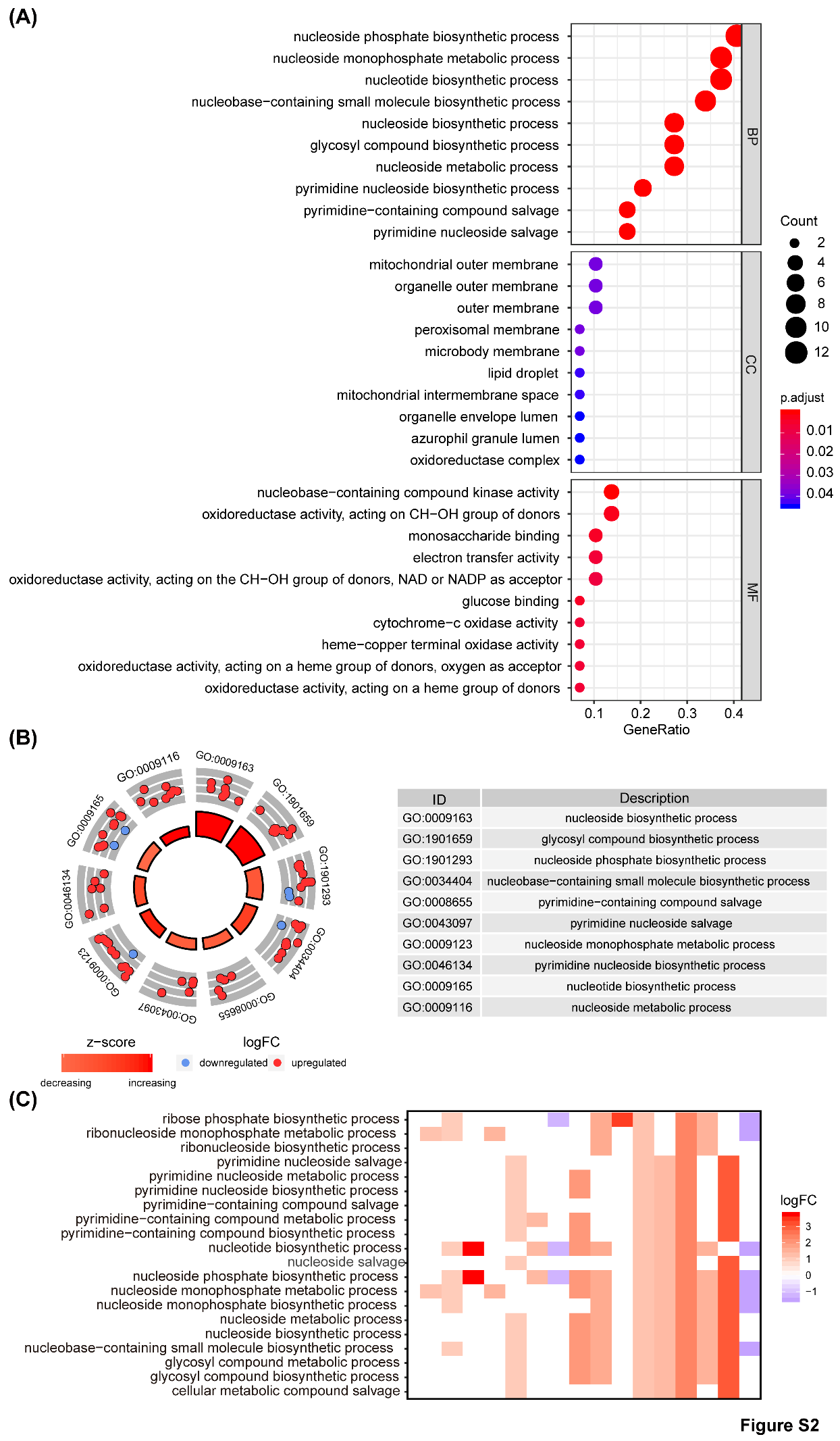


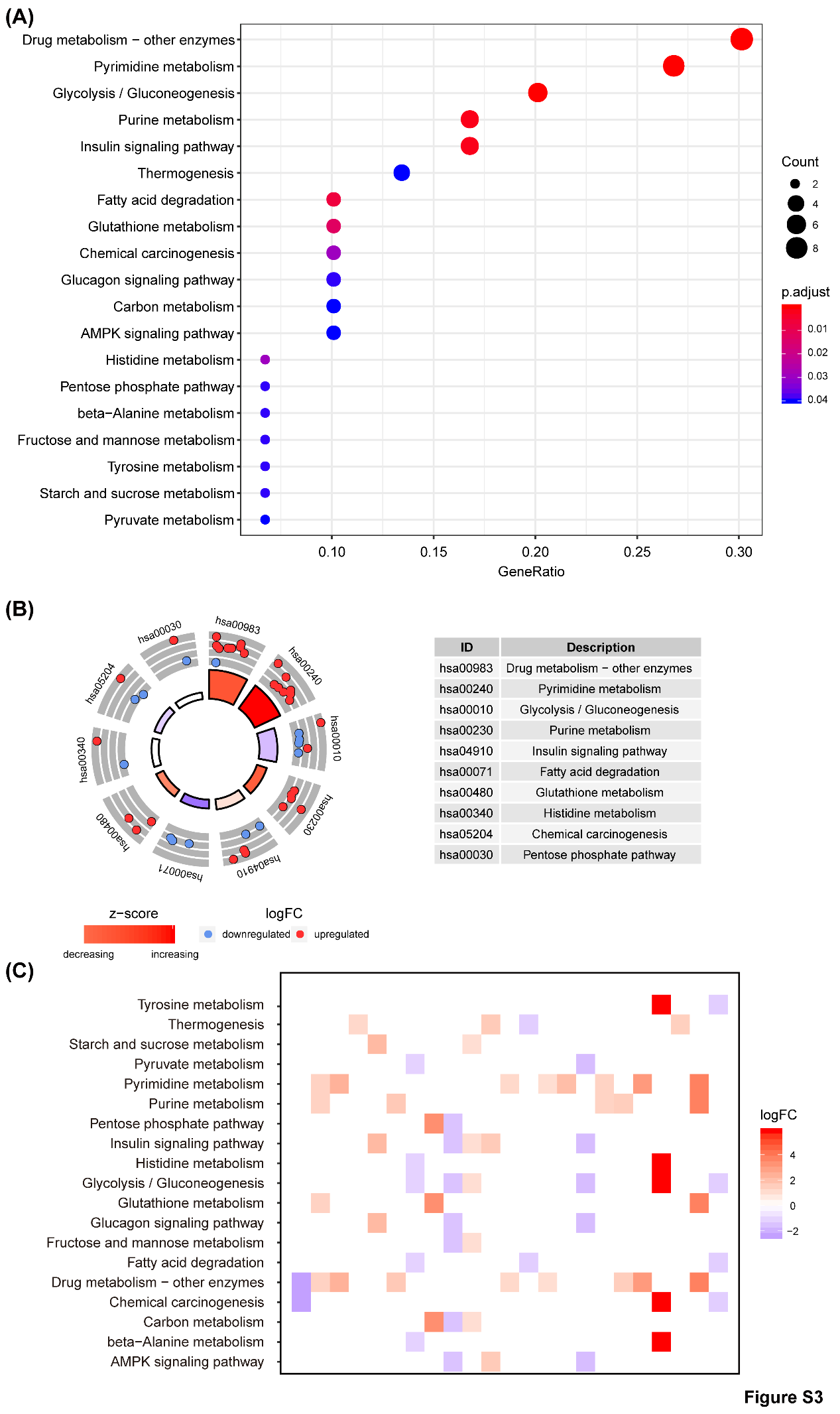


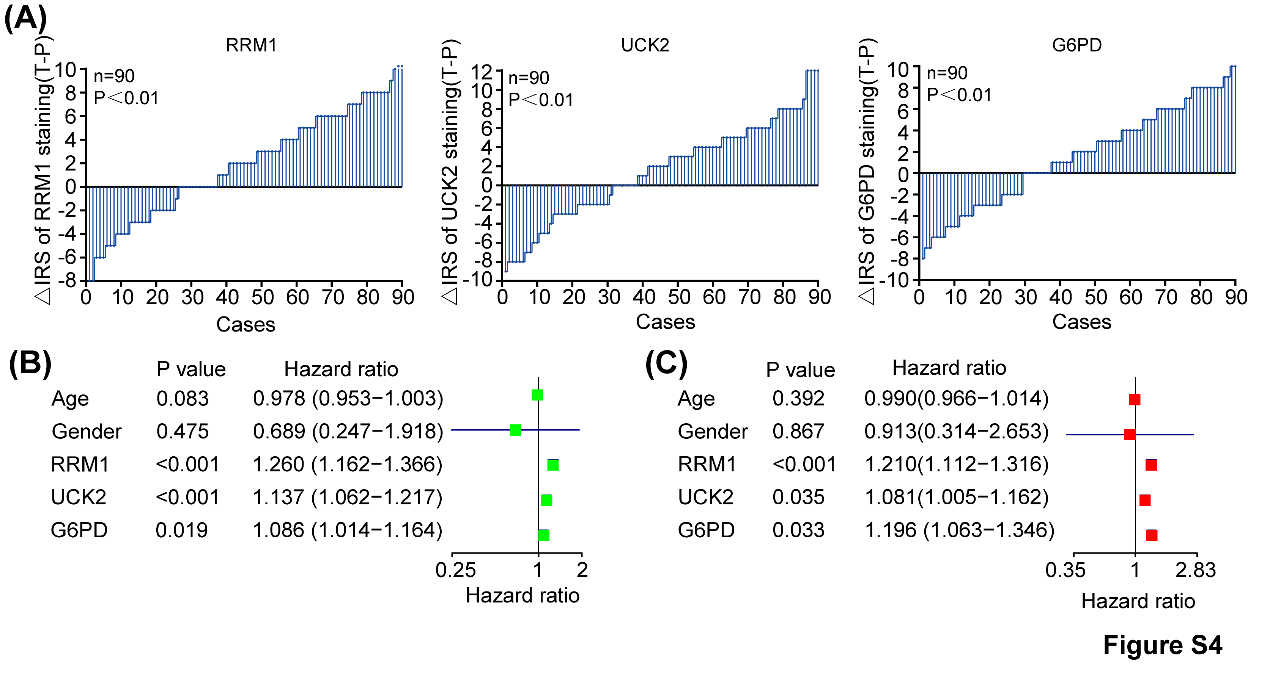


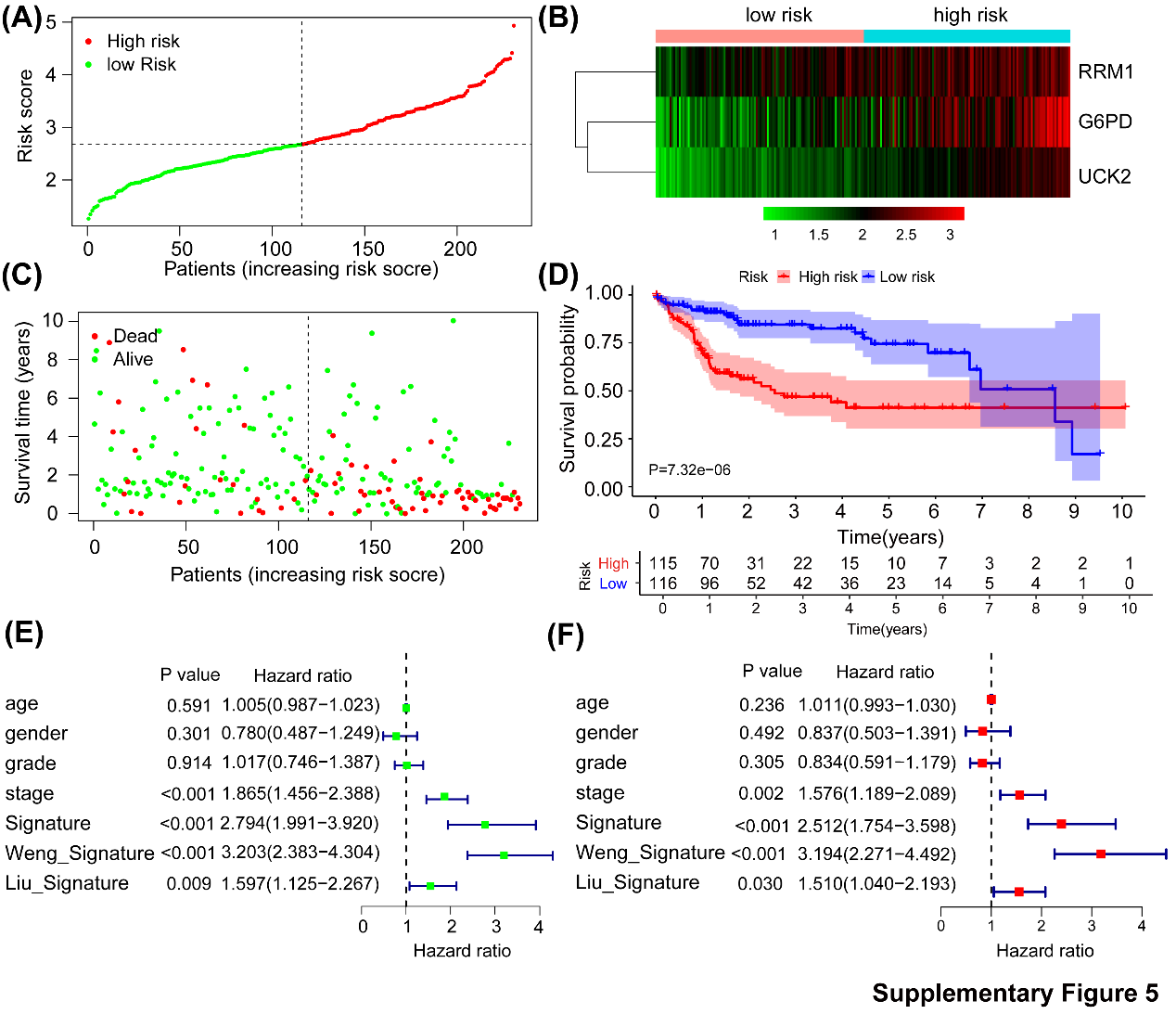


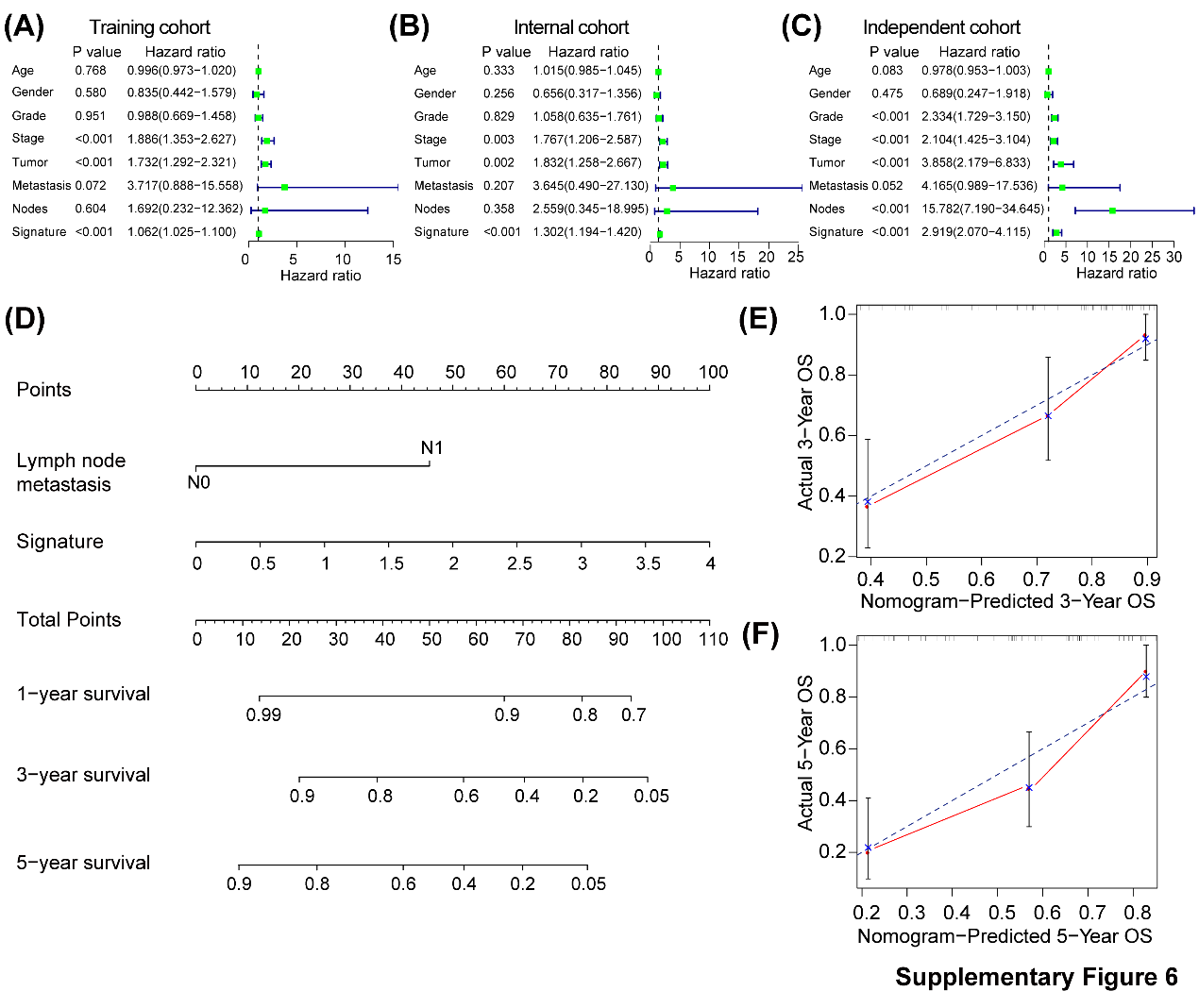

Supplement: Supplementary file 2 — Fig S1‐S6 [file CPR-54-e13117-s002.docx]
